# β-AR Blockers Suppresses ER Stress in Cardiac Hypertrophy and Heart Failure

**DOI:** 10.1371/journal.pone.0027294

**Published:** 2011-11-02

**Authors:** Li Ni, Changqing Zhou, Quanlu Duan, Jiagao Lv, Xiangning Fu, Yong Xia, Dao Wen Wang

**Affiliations:** 1 Departments of Internal Medicine, Tongji Hospital, Tongji Medical College, Huazhong University of Science and Technology, Wuhan, China; 2 Departments of Cardiothoracic Surgery, Tongji Hospital, Tongji Medical College, Huazhong University of Science and Technology, Wuhan, China; 3 The Davis Heart and Lung Research Institute, Division of Cardiovascular Medicine, Department of Molecular and Cellular Biochemistry, The Ohio State University College of Medicine, Columbus, Ohio, United States of America; Cardiovascular Research Institute Maastricht, Maastricht University, The Netherlands

## Abstract

**Background:**

Long-term β-adrenergic receptor (β-AR) blockade reduces mortality in patients with heart failure. Chronic sympathetic hyperactivity in heart failure causes sustained β-AR activation, and this can deplete Ca^2+^ in endoplasmic reticulum (ER) leading to ER stress and subsequent apoptosis. We tested the effect of β-AR blockers on ER stress pathway in experimental model of heart failure.

**Methods and discussions:**

ER chaperones were markedly increased in failing hearts of patients with **e**nd-stage heart failure. In Sprague-Dawley rats, cardiac hypertrophy and heart failure was induced by abdominal aortic constriction or isoproterenol subcutaneous injection. Oral β-AR blockers treatment was performed in therapy groups. Cardiac remodeling and left ventricular function were analyzed in rats failing hearts. After 4 or 8 weeks of banding, rats developed cardiac hypertrophy and failure. Cardiac expression of ER chaperones was significantly increased. Similar to the findings above, sustained isoproterenol infusion for 2 weeks induced cardiac hypertrophy and failure with increased ER chaperones and apoptosis in hearts. β-AR blockers treatment markedly attenuated these pathological changes and reduced ER stress and apoptosis in failing hearts. On the other hand, β-AR agonist isoproterenol induced ER stress and apoptosis in cultured cardiomyocytes. β-AR blockers largely prevented ER stress and protected myocytes against apoptosis. And β-AR blockade significantly suppressed the overactivation of CaMKII in isoproterenol-stimulated cardiomyocytes and failing hearts in rats.

**Conclusions:**

Our results demonstrated that ER stress occurred in failing hearts and this could be reversed by β-AR blockade. Alleviation of ER stress may be an important mechanism underlying the therapeutic effect of β-AR blockers on heart failure.

## Introduction

Heart failure remains a leading cause of hospitalization and death. A number of evidence indicates that the activity of sympathetic nervous system plays an important role in the pathogenesis of heart failure[1], [2], [3]. It has long been recognized that heart failure patients have higher catecholamine levels in their blood. Elevated circulating catecholamines result in chronic β-AR activation in the heart[4]. This has been thought to be a compensatory reaction to enhance cardiac contractility. Intriguingly, a number of clinical studies have demonstrated β-AR blockers as one of the few classes of drugs that improve cardiac function and reduce mortality in patients with heart failure[4]. But the mechanisms underlying the therapeutic effects of β-AR blockers on failing hearts have been poorly understood. One possible explanation is that β-blockers may compete with the binding of norepinephrine and epinephrine to their receptors, and thus attenuates the “fight or flight” reaction of the heart[5]. But it is unclear how blocking a pathway that is known to increases contractility of normal hearts can improve the function of failing hearts. Clearly, the putative mechanism by competing with the effect of catecholamines on cardiac contractility cannot fully explain the therapeutic effect of β-AR blockers on failing hearts. Other mechanisms likely exist. Elucidating these mechanisms will not only deepen the understanding on β-AR blockers therapy, it may also lead to new approaches to treat heart failure.

β-AR stimulation by elevated catecholamines activates dual signaling pathways mediated by the adenylate cyclase-cAMP-protein kinase A (PKA) and Ca^2+^/calmodulin-dependent protein kinase II (CaMKII)[6]. PKA phosphorylates and activates the ryanodine receptor RyR2 (sarcoplasmic reticulum Ca^2+^ release channel) [7]. CaMKII modulates an array of key proteins involved in Ca^2+^ handling, such as the sarcoplasmic/endoplasmic reticulum Ca^2+^-ATPase (SERCA) and its regulator, phospholamban (PLB), ryanodine receptor RyR2, and sarcolemmal L-type Ca^2+^ channels (LCC)[8]. Constant hyperactivation of RyR2 can lead to increased Ca^2+^ release and Ca^2+^ leak in myocytes during diastole[9], [10]. The long-term consequence of increased Ca^2+^ release and diastolic Ca^2+^ leak from the sarcoplasmic reticulum (SR) is the depletion of SR Ca^2+^ stores[9]. It is well established that Ca^2+^ depletion may induce ER stress[11]. ER stress is a series of adaptive responses in cells to alleviate the accumulation of unfolded proteins[12]. Normal protein folding requires adequate Ca^2+^ concentration in ER[13]. Ca^2+^ depletion, as occurred in failing cardiomyocytes, renders protein unfolding[13].

It has been reported that endoplasmic reticulum (ER) stress is involved in many heart diseases that contribute to heart failure at last, including artherosclerosis, myocardial ischemia, dilated cardiomyopathy[14], [15], [16]. The endoplasmic reticulum is a central organelle of each eukaryotic cell as the place of calcium storage, lipid synthesis, proteins folding and protein maturation[17]. Disturbances in any of these functions such as excessive accumulation of unfolded protein (unfolded protein response, UPR) or protein traffic can lead to the so-called ER stress[13]. The accumulation of unfolded proteins is sensed by three conserved pathways: IRE1α (inositol-requiring transmembrane kinase and endonuclease 1α), PERK (protein kinase-like ER kinase), and ATF6 (activation of transcription factor 6)[11]. Activation of these pathways stimulates an array of designated transcription factors (such as spliced XBP1, ATF6, and Activating Transcription Factor 4 [ATF4]), which subsequently trigger the expression of UPR-related genes (such as C/EBP homologous protein [CHOP] and Glucose-Regulated Protein 78 [GRP78])[12]. While the initial responses of these signaling pathways aim to assist protein folding, severe or prolonged ER stress will trigger the signals to apoptosis[13]. CHOP is an important component that mediates PERK activation-induced apoptosis in ER stress[18]. The c-Jun N-terminal kinase (JNK) is also activated in response to ER stress [19]. Indeed, ER stress-induced apoptosis has been shown to play important roles in the pathogenesis of diabetes and neurodegenerative disorders[20].

The chronic β-AR hyperactivation causes SR Ca^2+^ depletion, and this likely induces perpetual ER stress responses in failing heart. We thus hypothesize that ER stress is a critical downstream event of β-AR signaling pathway and β-AR blockers may protect cardiomyocytes by relieving ER stress. In the present study, we examined the hypothesis in cultured cells and animal models as well as in the human failing hearts. We found that chronic β-AR activation induces severe ER stress and apoptosis. β-AR blocker treatment markedly alleviates ER stress responses *in vitro* and *in vivo* leading to reduced hypertrophy and improved cardiac function. And the effect β-AR blockers on ER stress signaling may be contributable to inhibition of the CaMKII overactivation and restoration of the intracellular Ca^2+^ balance.

## Results

### Induction of ER Stress in Human Heart Failure

We collected heart samples from 4 receipts of heart transplantation who suffered from dilated cardiomyopathy with end-stage heart failure, and 9 patients undergoing mitral valve replacement, as well as 4 normal hearts of traffic accident victims. The candidate of patients undergoing mitral valve replacement presented with heart failure, including symptoms corresponding to a New York Heart Association (NYHA) class 3–4 heart failure (mean NYHA class 3.4±0.5) and left ventricular dilation on echocardiography (mean left ventricular end-diastolic diameter 57.6±10.2 mm at presentation) (Table 1).

**Table 1:** Clinical characteristics of patients with mitral valve replacement.

Patient	1	2	3	4	5	6	7	8	9	Summary
Age	43	27	58	63	42	52	45	56	48	48.2±10.7
Sex	male	female	female	female	female	female	female	female	female	N/A
Diagnosis	MI	MI	MS	MS	MI, TI	MVP	MS	MVP	MS	N/A
Diabetes	N	N	N	N	N	N	N	N	N	N/A
Hypertension	N	N	N	N	N	N	N	N	N	N/A
AF	N	N	Y	Y	N	N	N	N	N	2/9
NYHA	4	3	3	4	3	3	3	4	4	3.4±0.5
EF	78	66	48	63	56	57	45	55	50	57.6±10.2

MI, mitral insufficiency; MS, mitral stenosis; AF, atrial fibrillation; TI, tricuspid insufficiency; MVP, mitral valve prolapse; N/A, not applicable.

**Figure 1:**
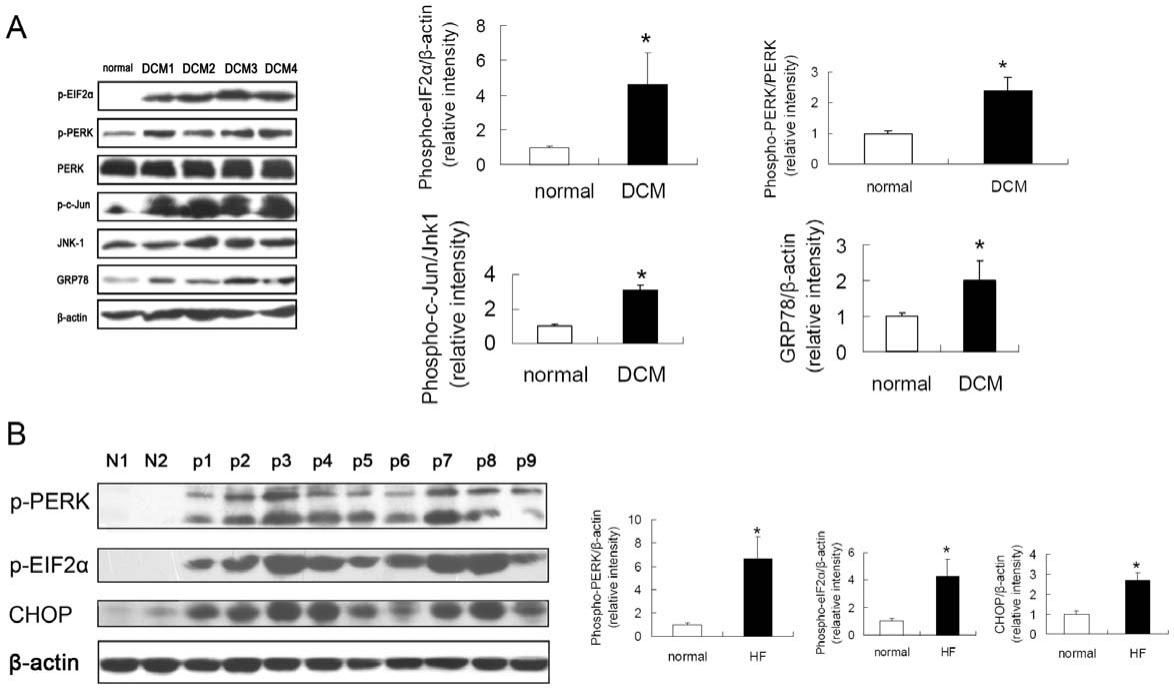
Induction of ER Stress in Human Heart Failure. ER stress markers including phosphorylated eIF2α (p-eIF2α), phosphorylated PERK (p-PERK), GRP78 and CHOP and apoptosis marker phosphorylated c-Jun (p-c-Jun), were examined in the heart samples from patients with heart failure. (A) ER stress was increased in heart transplant recipients’ failing hearts compared with normal heart. DCM, dilated cardiomyopathy. (B) p1-p9, heart samples of 9 patients undergoing mitral valve replacement; N1 and N2, normal human hearts. Proteins were normalized to β-actin. *P<0.05 vs. normal hearts.

We investigated the expression of several molecular indicators of ER stress in hearts of those patients, including GRP78, PERK, eIF2α and CHOP. We found significant activation of the PERK to eIF2α arm of the stress response in hearts from the patients with heart failure, evaluated by increased phosphorylated eIF2α and PERK compared with normal hearts (Figure 1A and 1B). Figure 1A also showed that JNK activity, indicated by c-Jun phosphorylation, was also significantly elevated in the human failure hearts. These results indicated that ER stress and its associated apoptosis signaling pathways appeared to be a general occurrence in human failing hearts arisen from varying diseases.

### β-AR Blockers Attenuated Cardiac Hypertrophy and Improved the Function of Failing Hearts

**Figure 2:**
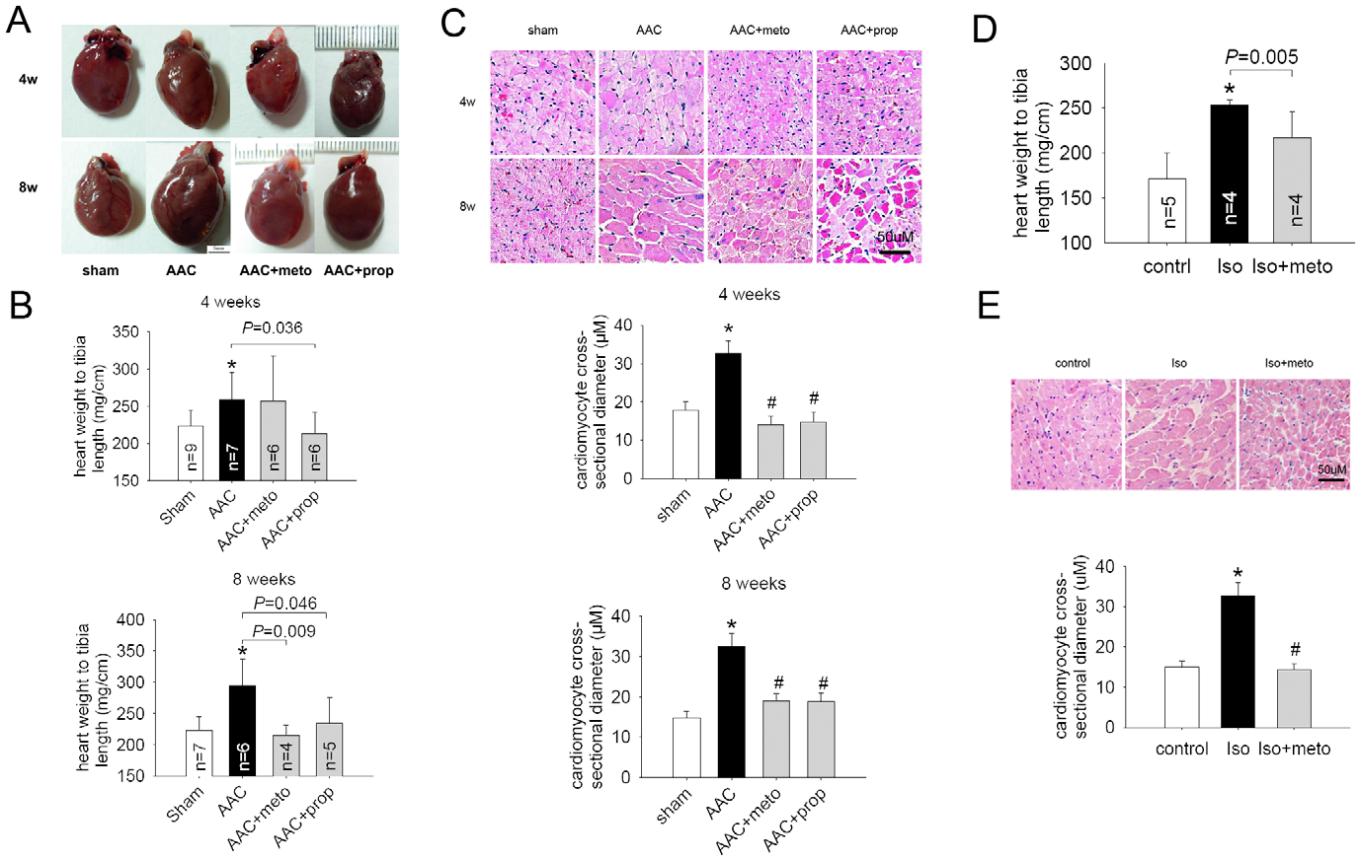
β-AR blockers attenuated cardiac hypertrophy and improved the function of failing hearts . (A) to (C) showed β-AR blockers attenuated cardiac hypertrophy in rats induced by abdominal aortic constriction (AAC). (A) Representative images of hearts. Scale, 5 mm. (B) Ratio of heart weight to body weight. *P<0.05 vs. sham. (C) HE staining of rat hearts and cardiomyocyte cross-sectional diameter (µm). Scale bar, 50 µm. *P<0.05 vs. sham, #P<0.05 vs. AAC. (D) and (E) showed β-AR blocker attenuated Iso-induced cardiac hypertrophy in rats. (D) Ratio of heart weight to body weight. *P<0.05 vs. control. (E) HE staining of rat hearts and cardiomyocyte cross-sectional diameter (µm). Scale bar, 50 µm. Iso, isoproterenol. *P<0.05 vs. control, #P<0.05 vs. Iso.

To investigate the effect of β-AR blockers on ER stress of failing hearts *in vivo*, we first established a cardiac hypertrophy model that shows cardiac dysfunction at chronic stage by performing an abdominal aortic constriction (AAC). The animals were divided into sham, AAC and β-AR blockers treatment groups. AAC-induced cardiac hypertrophy was assessed by increase of the ratio of heart weight to tibia length (HW/TL) and heart size. Metoprolol and propranolol treatments prevented cardiac hypertrophy induced by AAC (Figure 2A and 2B). Morphology analyses also showed that AAC induced a marked time-dependent increase myocyte hypertrophy, and this was significantly reduced in the hearts of β-AR blockers-treated rats (Figure 2C).

Detailed examination of heart function was carried out by invasive pressure-volume analysis. As shown in table 2, cardiac function in AAC rats was decreased in comparison with that sham animals as evidenced by the markedly reduced dP/dtmax and dP/dtmin, and increased left ventricular end diastolic pressure (LVEDP) and tau (table 2, *P*<0.01). Contractile function assessed through dP/dtmax was improved by β-AR blockers as compared to AAC rats; similar results were observed for diastolic function (LVEDP, tau and dP/dtmin) (Table. 2, *P*<0.05). Ventricular afterload (indexed by Ea, arterial elastance) was identically elevated by AAC and restored by administration of propranolol for 4 weeks (Table. 2, *P*<0.05).

**Table 2:** Hemodynamic parameters in AAC rats and AAC rats with β-AR blocker treatment.

	Sham	AAC	AAC+Meto-4w	AAC+ Prop-4w	AAC+Meto-8w	AAC+ Prop-8w
N	6	5	5	4	8	6
HR	189.3±9.9	195.6±19.4	208.8±28.5	200.5±23.2	202.6±21.3	207.5±11.5
LVEDP	0.4±3.3	4.9±2.6*	0.5±1.9†	−5.2±2.7†	8.1±3.1	5±3.1
Ea	1.45±0.85	2.19±0.7*	1.90±1.40	1.2±0.4†	1.70±1.20	1.54±0.4
dPdtMax	6516±1211	4448±876*	5147±627	6566±1401†	6169±1568†	6752±1064†
dPdtMin	−7211±1820	−5060±940*	−6077±2152	−8054±2463†	−7260±1839†	−7480±1507†
tauWeiss	14.3±2.8	17.9±2.3*	16.4±2.2	13.7±1.4†	22±4.6	17.5±1.4

HR, heart rate; LVEDP, left ventricular end diastolic pressure; Ea, arterial elastance; dP/dtmax, maximal slope of systolic pressure increment; dP/dt min maximal slope of diastolic pressure decrement; meto, metoprolol; prop, propranolol. Data are mean ± s.d. * P<0.05 versus control; †P<0.05 vs. AAC.

We carried out another animal model of heart failure with continuous infusion of β-adrenergic agonist isoproterenol (Iso) for 2 weeks, concurrently treated them with metoprolol or vehicle. Similar results were also observed from these Iso rats. Iso infusion induced significantly cardiac hypertrophy, and metoprolol treatment blunted the Iso-induced heart hypertrophy (Figure 2D and 2E). The left ventricle function was slightly improved assessed by invasive pressure-volume analysis (Table. 3). This data suggested that β-AR blockers attenuated cardiac hypertrophy and improved heart function in rats of heart failure.

**Table 3:** Hemodynamic parameters in Iso rats and Iso rats with β-AR blocker treatment.

	Control	Iso	Iso+meto
N	5	4	5
HR (bpm)	228.3±22.7	217.5±23.4	189.0±16.5
Pes (mmHg)	136.9±15.8	136.7±13.1	163.4±49.9
Ped (mmHg)	2.8±7.2	4.0±6.8	14.6±7.8
dP/dtmax (mmHg/s)	8134±1088	5933±800*	6305±1406
dP/dtmin (mmHg/s)	−7521.3±605	−5425±1912*	−5033±2190

HR, heart rate; PES, end-systolic pressure; PED, end-diastolic pressure; dP/dtmax, maximal slope of systolic pressure increment; dP/dt min maximal slope of diastolic pressure decrement; Iso, isoproterenol; meto, metoprolol. Data are mean ± s.d. *P<0.05 vs. control.

### β-AR Blockers Suppressed ER Stress in Hypertrophic and Failing Hearts of Rats

**Figure 3:**
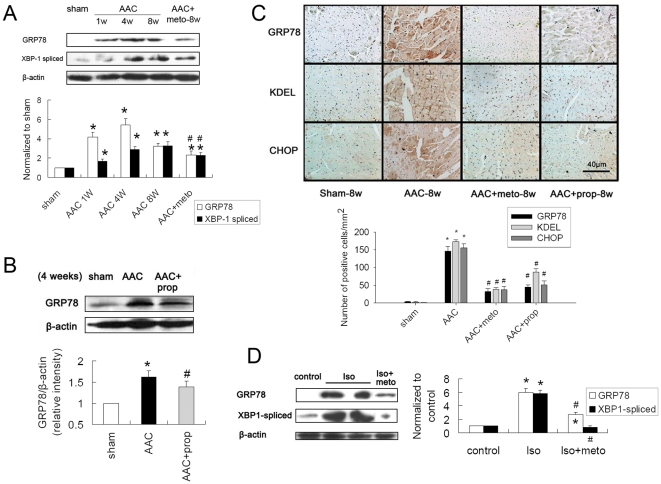
β-AR blockers suppressed ER stress in hypertrophic and failing hearts of rats. (A) ER chaperone GRP78 and spliced XBP-1 were increased in AAC rats (1or 4 or 8 weeks after operation), and metoprolol treatment (30 mg/kg/d for 8 weeks) reduced the epression of GRP78 and spliced XBP-1. Proteins were normalized to β-actin. (B) GRP78 was increased in AAC rats (4 weeks), and propranolol treatment (30 mg/kg/d for 4 weeks) reduced the epression of GRP78. Protein was normalized to β-actin. (C) Immunohistochemical analyses of rats’ hearts and number of GRP78, KDEL and CHOP-positive cells per mm^2^. Scale bar, 40 µm. For (A) to (C), *P<0.05 vs. sham, #P<0.05 vs. AAC. (D) GRP78 and spliced XBP-1 was increased in Iso rats, and metoprolol treatment (30 mg/kg/d for 2 weeks) reduced the epression of GRP78 and spliced XBP-1. Proteins were normalized to β-actin. *P<0.05 vs. control, #P<0.05 vs. Iso.

To explore the effect of β-AR blockers on ER stress in failing hearts in vivo, we examined the expression of several molecular indicators of ER stress in hypertrophic and failing hearts of rats. ER stress responses were seen in the process of cardiac hypertrophy and heart failure, as assessed by increased expression of GRP78 and spliced XBP-1 (Figure 3A and 3B). Metoprolol and propranolol therapy significantly suppressed the ER stress responses in hypertrophic and failing myocardium.

**Figure 4:**
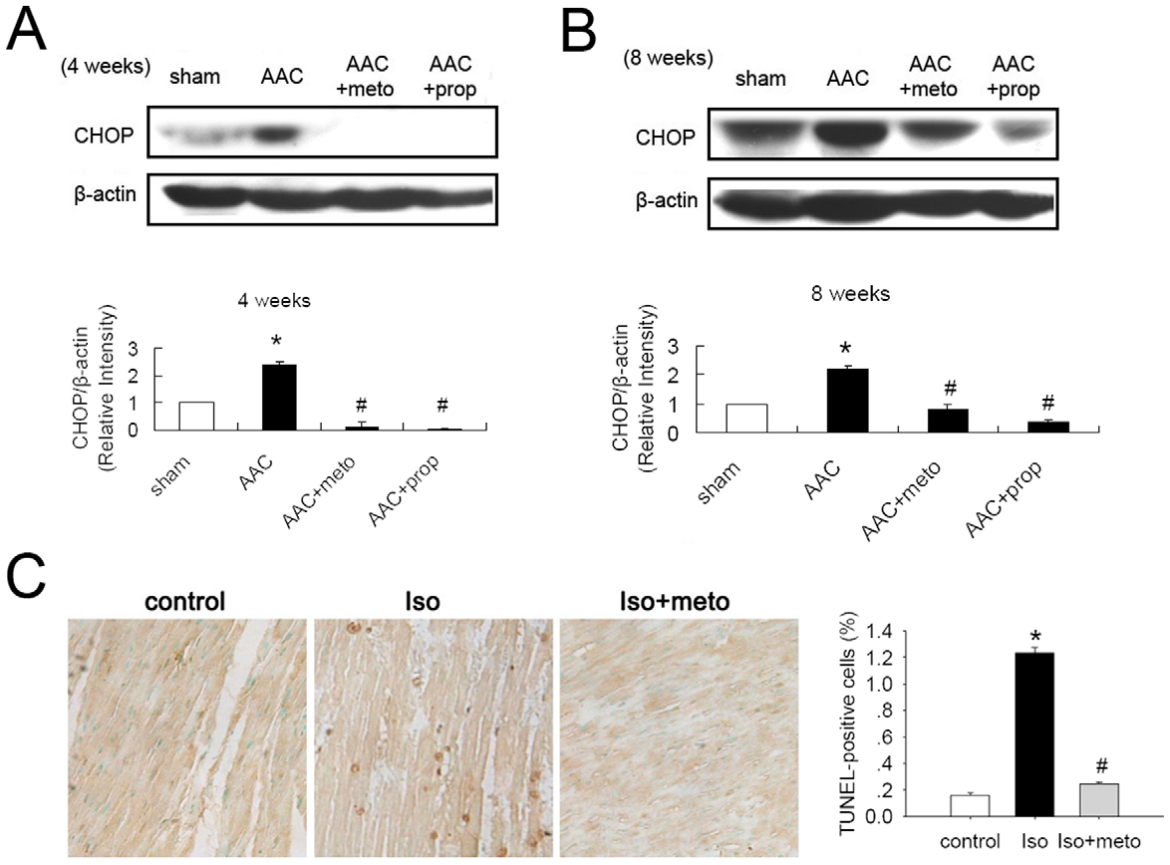
β-AR blockers suppressed ER stress-mediated apoptosis in cardiac hypertrophy and failure. (A) and (B) CHOP was increased in AAC rats (4 or 8 weeks after operation), and metoprolol (30 mg/kg/d for 4 or 8 weeks)or propranolol (30 mg/kg/d for 4 or 8 weeks) treatment reduced expression of CHOP. CHOP was normalized to β-actin. (C) Representative images of TUNEL showing cardiac myocytes apoptosis and quantitative analysis of TUNEL-positive myocardial cells in rats. Nuclei of normal cells are blue, and nuclei of apoptosis cells (TUNEL-positive cells) are brown.

To further support this notion, we also detected the effect of β-AR blockers on ER stress process through immunohistochemical analyses. As shown in Figure 4C, it revealed that the number of GRP78-positive cells, as well as KDEL -positive cells and CHOP-positive cells was increased in the hearts of the rats after AAC, and treatment with metoprolol or propranolol significantly reduced those positive cells.

Similar to the findings above, sustained Iso infusion for 2 weeks induced marked ER stress in hearts. β-AR blockers treatments suppressed ER stress responses in Iso-induced hypertrophic and failing hearts in rats (Figure 3D). These data exhibited the importance of increased catecholamines in the onset of ER stress in cardiac remodeling and failure. Taken together, all evidence above demonstrated that β-AR blockade alleviated ER stress in cardiac hypertrophy and heart failure in rats.

### β-AR Blockers Suppressed ER Stress-Mediated Apoptosis in Cardiac Hypertrophy and Failure

We examined CHOP expression in hearts subjected to AAC with or without β-AR blocker treatment. Results showed that the expression of CHOP increased dramatically after AAC; and metoprolol or propranolol treatment abolished expression of CHOP (Figure 4A and 4B). And treatment with metoprolol markedly decreased the number of apoptotic cells in the failing hearts of rats exposed to chronic Iso stimulation (Figure 4C). Thus, sustained ER stress triggered apoptosis in hypertrophic and failing hearts induced by either chronic loading stress or β-adrenergic stimulation, and these could be prevented by β-AR blockade.

### β-AR Blockers Reduced ER Stress in Cardiomyocytes

**Figure 5:**
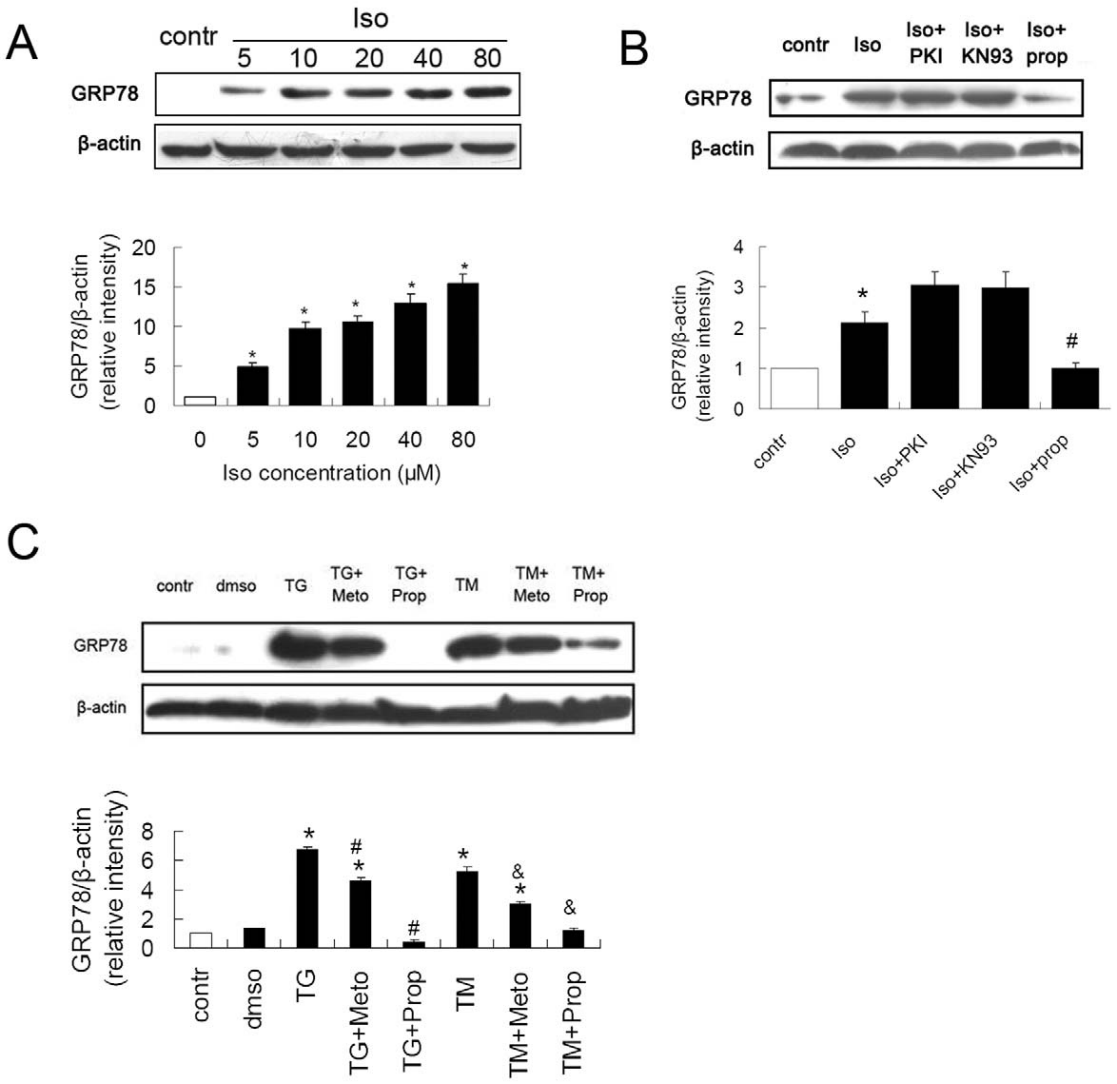
β-AR blockers alleviated ER stress induced in cardiomyocytes. (A) Iso increased GRP78 in a dose-dependent manner in H9c2(2–1) cells. *P<0.05 vs. control (B) Propranolol reduced Iso induced-ER Stress in H9c2(2–1) cells. Cells were pretreated with PKI (specific inhibitor of PKA, 5 µmol/L), KN-93 (specific inhibitor of CaMKIIδ, 0.5 µmol/L), metoprolol (meto, 10 µmol/L) or propranolol (prop, 10 µmol/L) for 1 hour, and exposed to Iso (10 µmol/L) for 24 hours. Cell lysates were then immunoblotted for GRP78, normalized to β-actin as a loading control. Iso, isoproterenol. *P<0.05 vs. control, #P<0.05 vs. Iso. (C) β-AR blockers blocked ER stress induced by TG or TM. Cells were treated with TG (5 µmol/L) or TM (5 µg/ml) with or without metoprolol (10 µmol/L) or propranolol (10 µmol/L) for 24 hours. Cell lysates were then immunoblotted for GRP78, normalized to β-actin. dmso, cell with vehicle dissolvant dimethyl sulphoxide; TG, thapsigargin; TM, tunicamycin. *P<0.05 vs. control, #P<0.05 vs. TM, & P<0.05 vs. TG.

To investigate whether the action of β-AR participate in ER stress, we pretreated H9c2(2–1) cells with Iso,and found that Iso significantly increased expression of GRP78 (Figure 5A). To investigate whether initiation of ER stress induced by β-AR stimulation is associated with PKA or CaMKII signaling pathway, we pretreated H9c2(2–1) cells with PKI (a specific inhibitor of PKA), KN93 (a specific inhibitor of CaMKIIδ) or propranolol before exposing them to Iso. Propranolol remarkably decreased Iso-induced GRP78 overexpression, while PKI, KN93 had no such significant effect (Figure 5B).

To reconfirm whether β-AR blockade inhibits the ER stress generally, we pretreated H9c2(2–1) cells with different β-AR blockers, metoprolol and propranolol, before exposing them to tunicamycin (TM) or thapsigargin (TG), agents commonly used to induce ER stress. Metoprolol and propranolol both significantly decreased TG or TM-induced GRP78 overexpression, especially, propranolol almost completely inhibited the TG-induced GRP78 activation after 24 hours incubation (Figure 5C). These results suggested that β-AR blockers reduced ER stress induced by different stressors in cells.

### β-AR Blockers Protected Cardiomyocytes against ER Stress-Mediated Apoptosis

**Figure 6:**
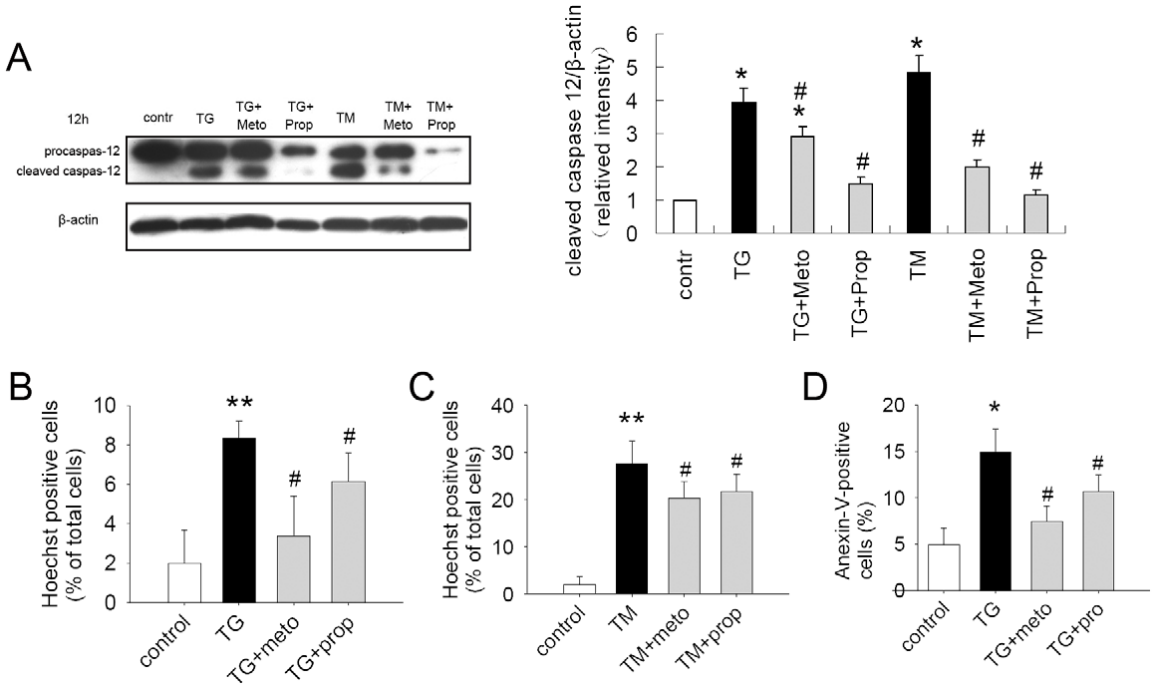
β-AR blockers protected cardiomyocytes against ER stress-mediated apoptosis. (A) β-AR blockers suppressed activation of caspase-12 in H9c2(2–1) cells. Cells were treated with TG (5 µmol/L) or TM (5 µg/ml) with or without metoprolol (10 µmol/L) or propranolol (10 µmol/L) for 12 hours. Cell lysates were then immunoblotted for caspase-12. Caspase-12 cleavage was normalized to β-actin. *P<0.05 vs. control, #P<0.05 vs. TM, & P<0.05 vs. TG. (B) and (C) Hochst-positive cells (%). H9c(2–1) cells were pretreated with metoprolol (10 µmol/L, 1 h) or propranolol (10 µmol/L, 1 h), then exposed to TG (5 µmol/L, 24 h) or TM (5 µg/ml, 24 h) before staining with Hochst33258 as indicated. Hochst-positive cells are expressed as a percentage of the number of total cells. **P<0.001 vs. control. # P<0.05 vs. TG or TM. (D) Anexin V-positive cells (%). H9c(2–1) cells were pretreated with metoprolol (10 µmol/L, 1 h) or propranolol (10 µmol/L, 1 h), then exposed to TG (5 µmol/L, 24 h) before staining with Anexin V/PI as indicated. Analyze by flow cytometry immediately after incubation. Anexin V-positive cells are expressed as a percentage of the number of total cells. *P<0.05 vs. control. #P<0.05 vs. TG.

Severe or prolonged ER stress triggers apoptosis. The characteristic markers of ER stress-induced apoptosis are c-Jun N-terminal kinase (JNK) and caspase-12 activation. As shown in Figure 6A, metoprolol and propranolol both reduced caspase-12 cleavage in H9c2 cells treated by TG or TM, and propranolol had the stronger effect which nearly blocked the activation of caspase-12. In accordance with results above, pretreatment of the cells with metoprolol or propranolol significantly reduced cell apoptosis induced by TG or TM as determined by Hoechst staining (Figure 6B and 6C) and Annexin V-FITC binding with flow cytometry analysis (Figure 6D). These results indicated that β-AR blockade reduced ER stress responses and subsequent apoptosis in cardiomyocytes.

### β-AR Blockers Suppressed Overactivation of Calmodulin Kinase II in Rat Failing Hearts

**Figure 7:**
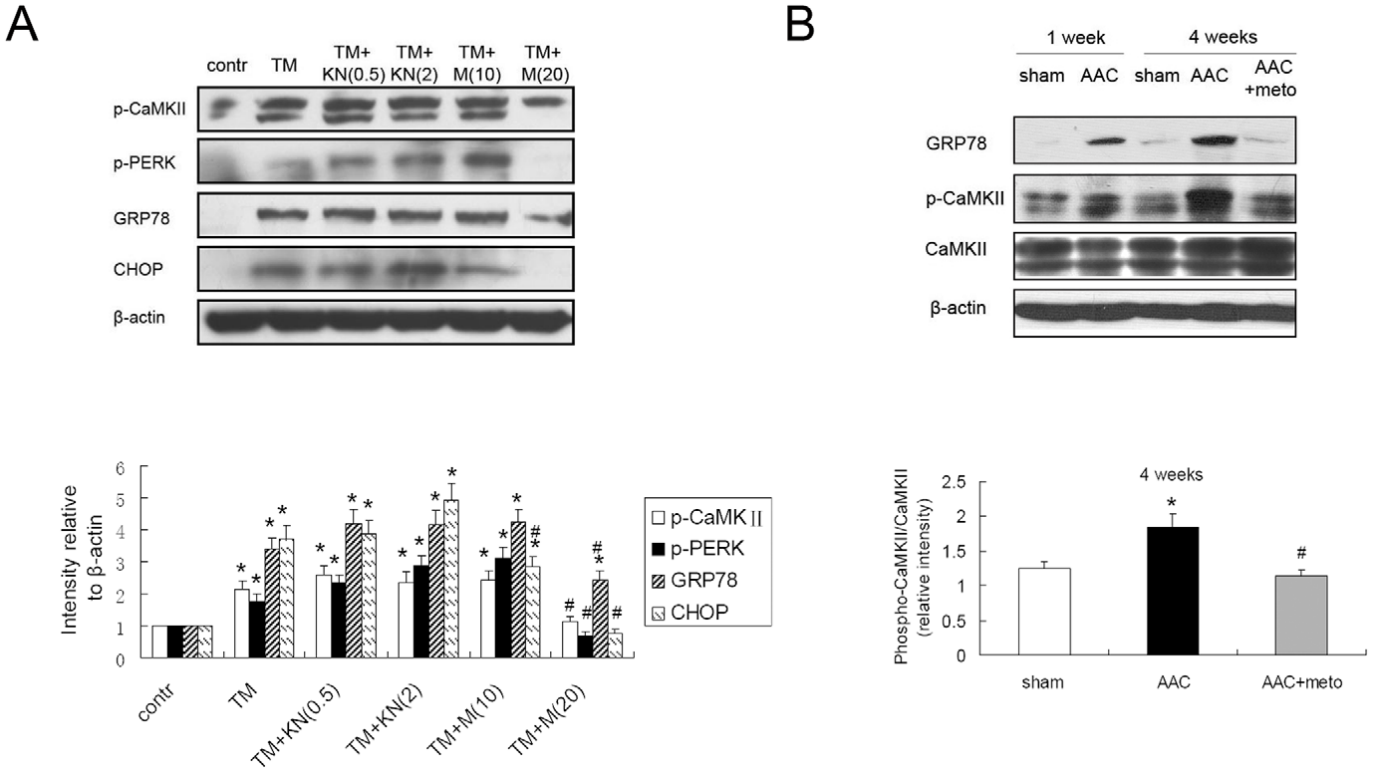
β-AR blockers suppressed overactivation of Calmodulin Kinase II in rat failing hearts. (A) Metoprolol alleviated ER stress and overactivation of CaMKII induced by tunicamycin (TM). Cells were pretreated with KN93 (KN, 0.5 µmol/L or 2 µmol/L), metoprolol (M, 10 µmol/L or 20 µmol/L) for 1 hour, and exposed to TM (5 µg/ml) for 24 hours. Cell lysates were then immunoblotted for phosphorylated CaMKII, phosphorylated PERK, GRP78 and CHOP, which were all normalized to β-actin. *P<0.05 vs. control, #P<0.05 vs. TM. (B) Metoprolol suppressed the overactivation of CaMKII in AAC rats. *P<0.05 vs. sham, #P<0.05 vs. AAC.

We pretreated H9c2(2–1) cells with KN93 or more selective β_1_-blocker metoprolol before exposing them to TM. Figure 7A showed specific inhibition of CaMKIIδ with KN93 did not suppress the ER stress, while metoprolol alleviated ER stress assessed by decreased expression of GRP78, phosphorylated PERK and CHOP. We assessed activation of CaMKII in rats failing hearts. As showed in Figure 7B, CaMKII significant activated in the AAC hearts, evaluated by increased phosphorylated CaMKII compared with normal hearts. And administration of metoprolol could suppress the overactivation of CaMKII. Taken together, these results suggest that β-AR blockade decreased overactivation of CaMKII, which could decrease intracellular Ca^2+^ and suppress ER stress.

## Materials and Methods

### Materials

Metoprolol, propranolol, thapsigargin (TG), tunicamycin (TM), isoproterenol (Iso), KN-93 were purchased from Sigma-Aldrich (St. Louis, MO). Antibodies for phosphorylated PERK, PERK, phosphorylated eIF2α, JNK1, phospho-c-Jun (p-Ser73), GRP78, CHOP (GADD153), KDEL receptor, XBP1, phosphorylated CaMKIIδ, CaMKIIδ, β-actin and PKI (6–22) were from Santa Cruz Biotechnology (Santa Cruz, CA). Antibody for caspase-12 was from Chemicon (Millipore, Billerica, MA). Horseradish peroxidase-conjugated secondary antibodies (goat anti-rabbit immunoglobulin G and rabbit anti-mouse immunoglobulin G) and enhanced chemiluminescence reagents were from Pierce Biotechnology (now part of Thermo Fisher Scientific, Rockford, IL). Polyvinylidene difluoride (PVDF) membranes were from Whatman (now part of GE Healthcare Life Sciences, Buckinghamshire, UK). Hoechst 33258 was from Calbiochem (part of Merck KGaA, Darmstadt, Germany) and Annexin V-FITC apoptosis detection kit and TACSTM TdT Kit apoptosis detection kit were from R&D (Minneapolis, MN). All other reagents were purchased from commercial suppliers unless otherwise specified.

### Cell Culture

H9c2(2–1) cells, a subclone of the original clonal cell line derived from embryonic BD1X rat heart tissue, were obtained from American Type Culture Collection (CRL-1446) and cultured in Dulbecco's modified Eagle's medium (DMEM) supplemented with 10% fetal bovine serum (FBS) and penicillin-streptomycin (100 IU/ml) in a humidified atmosphere of 95% air and 5% CO^2^ at 37°C.

### Animals

Male Sprague-Dawley rats (150–200 g) were obtained from the Experimental Animal Center of Tongji Medical College (Wuhan, People's Republic of China). All animal experimental protocols complied with the “Guide for the Care and Use of Laboratory Animals” published by the United States National Institutes of Health. The study was approved by the Institutional Animal Research Committee of Tongji Medical College (permit number: SYXK 2004–0028). All animals were housed at the animal care facility of Tongji Medical College at 25°C with 12/12-h light/dark cycles and allowed free access to normal rat chow and water throughout the study period. Rats were randomly assigned to different treatment groups.

### Experimental Groups

Pressure overload was induced by abdominal aorta constriction (AAC) in male Sprague Dawley rats according to the method described previously[21]. 80 rats were randomly assigned to one of 4 experimental groups: In group 1 (n = 20), sham-operated animals served as controls. In group 2 (n = 20), cardiac hypertrophy and heart failure was induced by abdominal aorta constriction (AAC) without further treatment. In group 3 (n = 20), AAC was performed and treatment with metoprolol for 4 or 8 weeks at a dose of 30 mg/kg/day in saline after operation as described previously[22]. In group 4 (n = 20), AAC was performed and treatment with propranolol for 4 or 8 weeks at a dose of 30 mg/kg/day in saline after operation.

Sustained β-adrenergic stimulation can be induced by low dose of isoproterenol (Iso) subcutaneous injection as reported before[23]. 48 rats were randomly divided in 3 groups: In group 1 (n = 16), rats were administered with saline subcutaneously per day as control. In group 2 (n = 16), rats were administered with Iso in saline subcutaneously 5 mg/kg/d for 2 weeks. In group 3 (n = 16), treatment with metoprolol in saline at a dose of 30 mg/kg/d was started when rats were exposing to Iso for 2 weeks.

### Abdominal Aorta Constriction

Cardiac hypertrophy and heart failure was induced by AAC according to the method described previously[21]. Briefly, rats were anesthetized with pentobarbital sodium at a dose of 40 mg/kg body weight intraperitoneally. Once anaesthesia is induced, it can be maintained by using a nose cone of halothane. The animal should be carefully monitored from the point of view of body temperature, respiratory rate, circulation, airway problems and injury from sharp objects for the adequacy of anaesthesia. And then the retroperitoneum was entered at 2 cm left lumbar vertebrae under the costal arch through a small incision. The abdominal aorta was isolated upper the renal artery crotch and constricted by a 4–0 silk suture ligature tied against a 22-gauge needle. The needle was removed to form a constriction of 0.7 mm in diameter. Sham-operated rats underwent a similar surgical procedure without aorta constriction. The animals were divided into sham receiving oral administration of saline, AAC rats with just saline as control, and treatment groups. In treatment groups, animals were received β-AR blocker metoprolol or propranolol for 4 or 8 weeks at a dose of 30 mg/kg/day in saline after operation as described previously[22].

### *In vivo* Hemodynamics

*In vivo* LV function was assessed by Millar PV catheter as described previously[24]. Rats were anesthetized as described above and placed on heating pads with core temperature maintained at 37°C. A microtip pressurevolume catheter (SPR-838; Millar Instruments, Houston, TX) was inserted into the right carotid artery and advanced into the left ventricle (LV) under pressure control. After stabilization for 20 min, the signals were continuously recorded at a sampling rate of 1,000/s using an ARIA pressure-volume conductance system (Millar Instruments) coupled to a Powerlab/4SP analog-to-digital converter (AD Instruments, Mountain View, CA) and a personal computer. All pressure-volume loop data were analyzed using a cardiac pressure-volume analysis program (PVAN3.6; Millar Instruments), and HR, LV end-systolic pressure (PES), LV end-diastolic pressure (PED), left ventricular end diastolic pressure (LVEDP), left ventricular end systolic pressure (LVESP), arterial elastance (Ea), tauWeiss, maximal slope of systolic pressure increment (dP/dt max) and diastolic pressure decrement (dP/dt min) were computed as described previously[25], [26].

### Human Heart Samples

This study was approved by the Review Board of Tongji Hospital and Tongji Medical College. The subjects recruited to the study provided written informed consent. The investigation conforms to the principles outlined in the Declaration of Helsinki. Tissue samples were obtained and kept frozen in liquid nitrogen and then stored at −80°C until use.

### Histochemical Analysis

Formalin fixed hearts were embedded in paraffin, sectioned into 4 µm slices, and stained with H&E. In order to determine the expression of ER stress chaperons, KDEL receptors were detected by immunohistochemical staining of formalin-fixed and paraffin-embedded heart sections. Briefly, after deparaffinization and rehydration, slides were incubated in 3% hydrogen peroxide for 10 min, PBS containing 10% of goat serum for 30 min and with a primary antibody to KDEL (overnight incubation at 4°C). After washing, sections were stained with a secondary biotinylated anti-rabbit antibody (Vector, CA) (room temperature; 30 min), followed by streptavidin-peroxidase (DakoCytomation, Denmark) (room temperature; 30 min). Subsequently the slides were incubated in DAB chromogen for 5 min at room temperature. Then the sections were counterstained with hematoxylin, and coverslipped.

The sections were examined with HAIPS-2000 Pathological Imagic Analysis System developed by the Ultrastructural Pathology Department of Tongji Hospital. Cardiac myocyte cross-sectional diameter and KDEL-positive cells per 1 mm^2^ were measured. The distance across the myocardial cell at its narrowest plane across the nucleus was measured in 75 cells from each LV (25 from the epicardium, 25 from the myocardium, and 25 from the endocardium). The mean diameter was calculated for the LV in each animal. We counted the number of KDEL positive cells (brown staining) per mm^2^ for 5 different visual fields in each animal, and the average number was calculated in each animal and each group.

### Apoptosis Detection

#### Hoechst Staining

Hoechst33258 suspended in dH_2_O at a 1 mM concentration was prepared as stock solution. Cultured cells were suspended at approximately 1–2×10^6^/ml in buffered media (pH 7.2). Hoechst33258 dye was added to cell suspension to 10 µmol/L final concentrations, and incubated at 37°C for 30 minutes. The cells then were observed under fluorescence microscope or analyzed by flow cytometry immediately. Hoechst33258 stained cells were illuminated with an argon laser tuned for UV (346–352 nm) and resulting fluorescence were detected at 460 nm.

#### Annexin V-FITC Apoptosis Assay

The cultured cells were gently trypsinized and washed with serum-containing media. After centrifugation, cell pellets (1–5×10^5^ cells) were resuspended in 500 µl of 1x Binding Buffer, and 5 µl of Annexin V-FITC and 5 µl of propidium iodide (PI 50 µg/ml) were added. Annexin V-FITC binding was analyzed by flow cytometry (Ex  = 488 nm; Em  = 530 nm) using FITC signal detector (usually FL1) and PI staining by the phycoerythrin emission signal detector (usually FL2) immediately after 5 min incubation at room temperature in the dark.

#### TUNEL

Terminal deoxynucleotidyl transferase–mediated dUTP nick-end labeling (TUNEL) reaction was performed by using the TACSTM TdT Kit apoptosis detection kits (R&D).

### Immunoblotting

Cell lysates and lysates from heart tissues, prepared as previously described[27], were matched for protein concentration, separated on SDS polyacrynamide gels (8–12%) and transferred to nitrocellulose membranes. Next, the membranes were blocked with 5% nonfat milk and 3% BSA for 2 hour and incubated overnight with primary antibodies as indicated. The following day, membranes were washed three times and incubated with appropriate secondary antibody for 2 hour at room temperature. Antibody binding was detected by enhanced chemiluminescence.

### Statistical Analysis

All values are expressed as mean ± s.e. unless noted otherwise. Differences between data groups were evaluated for significance using Student *t*-test of unpaired data or one-way analysis of variance (ANOVA) and Bonferroni post-test. P<0.05 was accepted as statistically significant.

## Discussion

In the present study, we identify long-term oral β-AR blockers suppresses ER stress in cardiac hypertrophy and heart failure. We found the clue first, that ER stress was induced in the tissues from human failing hearts. The similar result was subsequently found in isoproterenol-stimulated cardiomyocytes and rat models of heart failure after abdominal aortic constriction and isoproterenol subcutaneous injection. β-AR blockers treatment reduced ER stress and associated apoptosis in cardiomyocytes and rats failing hearts. This effect may be attributable to prevention of CaMKII overactivation and restoration of intracellular Ca^2+^ balance.

Heart failure (HF) is a complex clinical syndrome that involves a series of responses in the heart, neural and hormonal systems, and vasculature[28]. It has been documented that ER stress is involved in cardiotoxicity and heart diseases that eventually may evolve into heart failure[14], [15], [16]. For instance, imatinib, the causal agent in chronic myelogenous leukemia, exhibit cardiotoxicity; and the underlying mechanism seems to be related to the ER stress caused by this drug[29]. ER can generate and propagate apoptotic signals in response to myocardial ischemic stress[15], [30]. Pressure overload by transverse aortic constriction induces expression of ER chaperones and ER stress-induced apoptosis of cardiac myocytes, leading to cardiac hypertrophy and heart failure[14]. Okada and his colleage found ER stress induced in patients with dilated cardiomyopathyv (DCM) and adriamycin cardiomyopathy[14]. In the present study, we found upregulation of ER chaperones in patients with end-stage heart failure, who received heart transplantation and mitral valve replacement. We detected the activation of several ER stress-associated pro-apoptotic signaling pathways, including PERK, eIF2α, CHOP and JNK, in human failing hearts. These results confirmed previous studies and indicated that prolonged ER stress and associated apoptosis appeared to be a general occurrence in human failing hearts arisen from varying diseases.

While previous studies implicated ER stress induced in heart failure, how ER stress is initiated in failing hearts is still unknown. Our present studies suggest that β-AR hyperactivation may be an important mechanism underlying ER stress in cardiac hypertrophy and failure. Sympathetic nervous system activity plays a central role in the pathogenesis of heart failure. Catecholamines are released from the sympathetic nervous system as a result of increased workload from pressure or volume overload[31]. The sustained increase in adrenoceptor activity initiates impairment in β-AR signaling pathway[32]. The identified important downstream effect of altered β-AR signaling in heart failure include hyperposphorylation of LTCC, the NCX, and cardiac ryanodine receptors[9], [33]. Chronic PKA hyperphosphorylation of RyR2 in heart failure leads to depletion of calcium in SR, which in turn reduces EC coupling gain and contributes to impaired systolic contractility[9], [10]. On the other hand, Ca^2+^ storage and signaling, as well as the folding, modifying and sorting of newly synthesized proteins, are among the main functions of the ER in mammalian cells[11]. Disturbances in any of these functions can result in ER stress[13]. Both Ca^2+^ overload and depletion of the ER Ca^2+^ pool can lead to changes in protein folding and in ER stress[11].

Indeed, we detected strong ER stress response in isoproterenol-stimulated cardiomyocytes and the hearts of rats subjected to constant β-AR stimulation. While these initial responses may be adaptive, prolonged ER stress triggers apoptotic signaling. Parallel to the activation of ER stress signaling pathways, apoptosis and loss of heart function were seen in cardiac hypertrophy and failure. These findings suggest that ER stress maybe a crucial downstream event of chronic β-AR hyperactivation and ER-induced apoptosis may be important cause of the loss of cardiac function in heart failure.

β-AR blockade is one of the most effective treatments with improving cardiac function and prolonging life in patients with heart failure[4]. However, the use of β-AR blockers in patients with heart failure is counterintuitive, because they decrease contractility acutely in normal and failing hearts. Current understanding on the mechanism of β-AR blockers therapy includes the attenuation of chronic hyperactivity of the sympathetic nervous system[5]. However, this does not fully explain the beneficial effect of β-AR blockers on failing hearts. The present study suggested that alleviation of ER stress may contribute to the action of β-AR blockers. In cultured cardiomyocytes, β-AR blockers prevented isoproterenol-induced ER stress responses. Furthermore, experimental therapies with β-AR blockers also attenuated ER stress reactions in hypertrophic and failing rat hearts. Corresponding to the attenuation of ER stress, pro-apoptotic signaling and the number of apoptotic cells were all diminished by β-AR blockers treatment. The preventative effect of β-AR blockers on ER stress is also parallel to the reduction of cardiac hypertrophy and improvement of cardiac function.

Moreover, the effect of nonselectived β-AR blocker propranolol on suppressing ER stress seemed stronger than selectived β_1_-AR blocker metoprolol. Previous study have ever indicated that propranolol treatment attenuates LVH by a mechanism unrelated to its β-adrenoceptor blocking activity, and is possibly mediated through the known membrane stabilizing effect of this agent[21]. Consider with metoprolol and propranolol had the same effect on β-AR blocking activity[22], our data above also suggested that the effect of propranolol on suppressing ER stress may be independent of its β blockade. Taken together, all lines of evidence indicate that the therapeutic effect of the β-AR blockers on heart failure may be, at least partially, attributed to their ability to counteract ER stress.

How exactly β-AR blockers prevent ER stress responses remains to be determined. One plausible explanation is that the administration of β-AR blockers reduces the chronic hyperactivity of the sympathetic nervous system. Deceased plasma levels of circulating catecholamines reduce intracellular cAMP levels, and thus reverse PKA hyperphosphorylation of RyR2[10], [34]. This may restore normal intracellular Ca^2+^ handling and Ca^2+^ signaling in failing heart. However, β-AR blockers also dramatically attenuated ER stress induced by TG and TM, which directly perturb ER Ca^2+^ homeostasis and protein glycosylation. ER stress induced by these two means is independent of β-AR activation. The fact that β-AR blockers attenuated ER stress induced by adrenergic stimulation independent means is a strong indication that β-AR blockers can alleviate ER stress by mechanisms independent of β-AR blockade.

Acute β_1_-adrenergic receptor (β_1_AR) stimulation activates the classic Gs-adenylyl cyclase (AC)-cAMP-PKA signaling pathway, whereas chronic stimulation of β_1_AR that induces myocyte hypertrophy and apoptosis requires activation of CaMKII[6], [35]. However, inhibition of PKA could not alleviate ER stress. It revealed that chronic β-adrenergic stimulation induced-ER stress may be independent of the typical PKA pathway. Increased activitation of CaMKII, a Ca^2+^- dependent kinase, elevates intracellular Ca^2+^. Previous studies[36], [37] have demonstrated that increase of intracellular Ca^2+^ was a common mechanism for aberrant ER stress and unfolded protein response activation. We found significant activation of CaMKII in isoproterenol-stimulated cardiomyocytes and rats failing hearts. And β-AR blockade suppressed overactivation of CaMKII, which could decrease intracellular Ca^2+^ and suppress ER stress. We suppose that effect of β-AR blockers on ER stress signaling may be contributable to inhibition of the CaMKII overactivation and restoration of the intracellular Ca^2+^ balance.

In summary, we demonstrated that ER stress is a crucial downstream event of β-AR hyperactivation in cultured cells and heart failure in vivo. β-AR blockade markedly relieved ER stress and ER mediated-apoptosis in cardiomyocytes and hypertrophic and failing hearts. Thus, alleviation of ER stress may be an important mechanism underlying the therapeutic effect of β-AR blockers on heart failure. Preventing or alleviating ER stress may represent a new approach to treat cardiac hypertrophy and heart failure.
